# Charge and Lattice Fluctuations in Molecule-Based Spin Liquids

**DOI:** 10.1038/s41598-017-13118-4

**Published:** 2017-10-10

**Authors:** Takashi Yamamoto, Takashi Fujimoto, Toshio Naito, Yasuhiro Nakazawa, Masafumi Tamura, Kyuya Yakushi, Yuka Ikemoto, Taro Moriwaki, Reizo Kato

**Affiliations:** 1 Graduate School of Science and Technology, Ehime University, 2-5 Bunkyo-cho, Matsuyama, 7908577 Japan; 2 RIKEN, 2-1 Hirosawa, Wako, 3510198 Japan; 3 Graduate School of Science, Osaka University, 1-1 Machikaneyama, Toyonaka, Osaka 560-0043 Japan; 4 Department of Physics, Faculty of Science and Technology, Tokyo University of Science, 2641 Yamazaki, Noda, 2788510 Japan; 5 Toyota Physical and Chemical Research Institute, 41-1 Yokomichi, Nagakute, 4801192 Japan; 6 JASRI, SPring-8, 1-1-1 Kouto, Sayo, Hyogo 679-5198 Japan

## Abstract

Spin liquid (SL) systems have been the subject of much attention recently, as they have been theoretically predicted to not freeze, even at 0 K. Despite extensive searches being made for such a system, only a few candidates have been found. All of these candidates share geometrical frustrations that are based on triangular lattices. We applied vibrational spectroscopy to one of the candidates of a molecule-based SL system, and we compared its results against three antiferromagnetic compounds and four charge-ordered compounds. All of their structural motifs belong to triangular lattices. The C=C stretching modes in the SL state indicated that there were charge and lattice fluctuations. These fluctuations were suppressed but non-negligible in the AF compounds. This finding is potentially significant, as it indicates that a hidden lattice and charge fluctuation are the driving force of a geometrical frustration, which eventually leads to a SL state.

## Introduction

**Figure 1:**
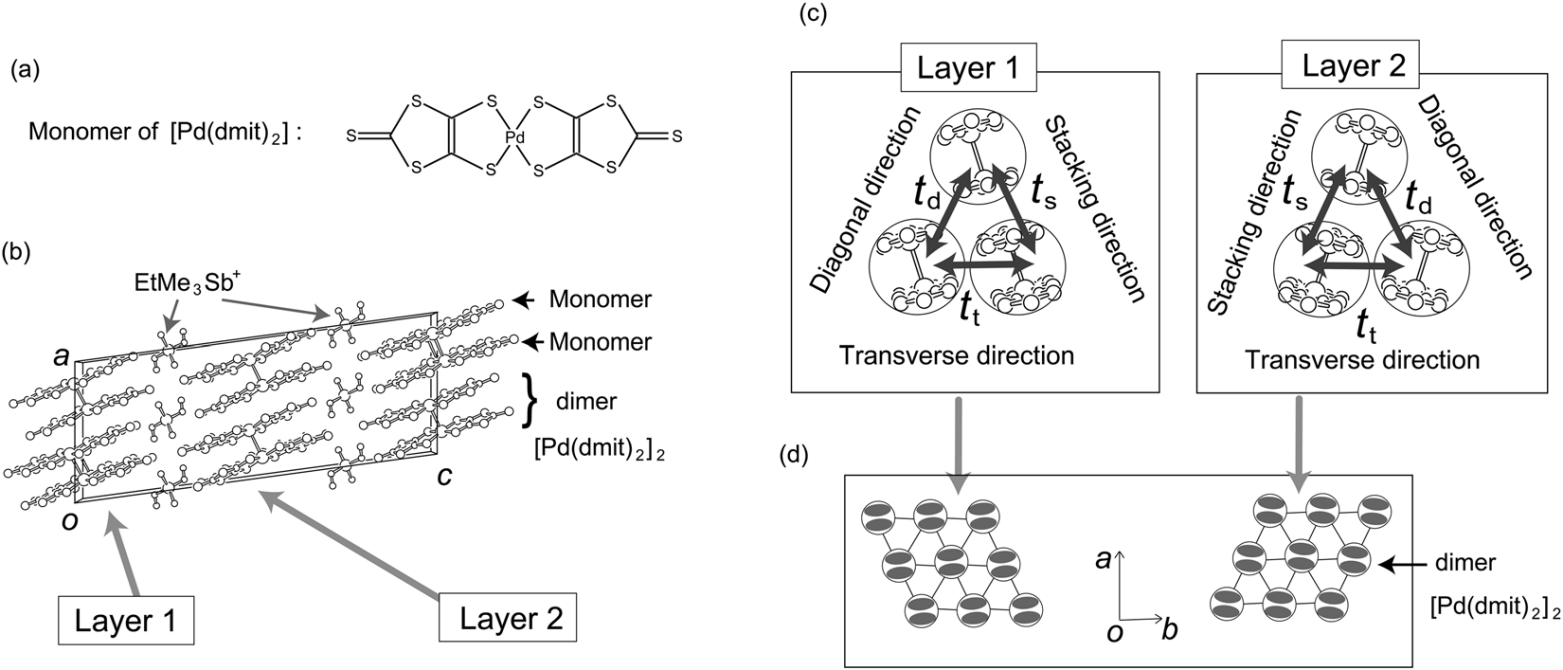
Molecular structure, crystal structure and schematic views of two-dimensional layers of *β*′–EtMe_3_Sb[Pd(dmit)_2_]_2_ (Sb-salt 2). (**a**) Monomer of Pd(dmit)_2_. (**b**) Crystal structure as viewed along the *b*-axis. (**c**) Two equivalent conducting layers with different orientations; layer 1 and layer 2. *t*_d_, *t*_s_ and *t*_t_ denote the transfer integrals between neighboring dimers in the diagonal, stacking and transverse directions, respectively. (**d**) Schematic views of the two-dimensional layers.

It has been more than 40 years since Anderson predicted quantum spin liquid (SL) states using his resonating–valence bond model^1^. These SL states have attracted much attention, as they are theoretically expected to not freeze, even at 0 K. In the last decade, efforts have been made to discover an actual example of a quantum SL state in triangular, Kagomé and pyrochlore lattices^2^. All of these lattice types are based on a triangular arrangement of the spin sites, which makes them incompatible with antiferromagnetic ordering. These triangular lattices hinder any ordering pattern of the spin arrangements being made when the spins are affected by antiferromagnetic interactions, which leads to geometrical frustration. It is within this context that some two-dimensional (2D) molecular solids have attracted a lot of interest, as their spin systems possess triangular lattices with *S* = 1/2^3–13^; examples of such solids include *β*′-EtMe_3_Sb[Pd(dmit)_2_]_2_ [dmit = 1,3-dithiole-2-thione-4,5-dithiolate], *κ*-(ET)_2_M_2_(CN)_3_ [ET = bis(ethylenedithio)tetrathiafulvalene, M = Cu and Ag] and *κ*-H_3_(Cat-EDT-TTF)_2_ [ = catechol-fused ethylenedithiotetrathiafulvalene]^14–23^. The triangular lattices of *β*′-EtMe_3_Sb[Pd(dmit)_2_]_2_ are comprised of a pair of independent monomers (Fig. 1(a)), where each pair is termed a dimer (Fig. 1(b–d))^16,24,25^. If one is to assume that an electron or a hole should be located at a dimer, then *β*′-EtMe_3_Sb[Pd(dmit)_2_]_2_ should have a half-filled band, which would lead to a Mott insulator. The structures of the triangular lattices of *κ*-(ET)_2_M_2_(CN)_3_ and *κ*-H_3_(Cat-EDT-TTF)_2_ are similar to that of *β*′-EtMe_3_Sb[Pd(dmit)_2_]_2_. The results of magnetic and transport property measurements have revealed the absence of any ordering, even at low temperature, which suggests that a spin liquid (SL) state occurs in *β*′-EtMe_3_Sb[Pd(dmit)_2_]_2_, *κ*-(ET)_2_M_2_(CN)_3_ and *κ*-H_3_(Cat-EDT-TTF)_2_^14–23^. However, a series of X[Pd(dmit)_2_]_2_ [X: a monovalent cation], whose 2D layer is isostructural to that of *β*′-EtMe_3_Sb[Pd(dmit)_2_]_2_, has been shown to have various ground states as shown in Table 1 ^16,24^. Hereafter, we use the abbreviations in Table 1. Similar variety is also observed in the *κ*-type compounds^5,18,26^.

**Table 1:** Ground states of X[Pd(dmit)_2_]_2_ salts and deviation from equilateral triangular lattices.

Material	Abbreviation of material	Ground state^*1^	*T*_AF_ or *T*_CO_ (K)^*2^	Calculated value of 2*t*_d_/(*t*_s_ + *t*_t_) at 300 K^*3^
*β*′–Me_4_P[Pd(dmit)_2_]_2_	P-salt 1	AF	*T*_AF_ = 42^21^	0.62^25^
*β*′–Et_2_Me_2_P[Pd(dmit)_2_]_2_	P-salt 2	AF	*T*_AF_ = 17^21^	0.84^25^
*β*′–Me_4_Sb[Pd(dmit)_2_]_2_	Sb-salt 1	AF	*T*_AF_ = 16^21^	0.86^25^
*β*′–EtMe_3_Sb[Pd(dmit)_2_]_2_	Sb-salt 2	SL	—	0.91^25^
*β*′–Et_2_Me_2_Sb[Pd(dmit)_2_]_2_	Sb-salt 3	CO	*T*_CO_ = 70^30^	1.01^30^
*monoclinic*−EtMe_3_P[Pd(dmit)_2_]_2_	*m*-salt	CO	*T*_CO_ = 20^49,50^	1.05^38^
*triclinic*−EtMe_3_P[Pd(dmit)_2_]_2_	*t*-salt	CO	*T*_CO_ = 50^39^	0.29^39^
*β*′–Cs[Pd(dmit)_2_]_2_	Cs-salt	CO	*T*_CO_ = 56^29^	0.74^30^

Abbreviations of the materials are also used in Fig. 3. ^*1^AF: antiferromagnetic state, SL: spin liquid state and CO: charge-ordered state. ^*2^ *T*_AF_ and *T*_CO_ denote the temperatures of the antiferromagnetic and charge-ordering phase transitions, respectively. ^*3^The transfer integral is estimated from an extended Hückel calculation using the atomic parameters from an X-ray structural analysis. The definitions of *t*_d_, *t*_s_ and *t*_t_ are shown in Fig. 1(c). The value of the equilateral triangular lattice is unity.

There is a significant difference between the molecular arrangements in the 2D layers of X[Pd(dmit)_2_]_2_ and *κ*-type compounds. The 2D layers of *κ*-type compounds always consist of isosceles or equilateral triangular lattices^18^, while the 2D layers in X[Pd(dmit)_2_]_2_ can be equilateral, isosceles or scalene lattices, depending on X^17,25^. Therefore, there is a variety of 2D layers that X[Pd(dmit)_2_]_2_ can have, whereas the 2D layers of *κ*-type compounds are specific. An additional important difference between them is that the inter- and intra-dimer transfer integrals are significantly smaller and larger, respectively, in X[Pd(dmit)_2_]_2_ than in *κ*-type compounds^24^. A large intra-dimer transfer integral is due to the chemical bond between monomers in a dimer, the result of which is an inversion in the energy levels of the molecular orbitals near the Fermi level *ε*_F_^27–29^. This energy inversion favours several kinds of charge-ordered (CO) states^30–40^.

As shown in Table 1, the antiferromagnetic (AF) transition temperature, *T*_AF_, decreases as the 2D layers become shaped more like equilateral triangular lattices^16,21,24,25^. The trend in Table 1 suggests that the magnetic ordering in *β*′-EtMe_3_Sb[Pd(dmit)_2_]_2_ (Sb-salt 2) is suppressed by a geometrical frustration. The specific heat capacity, thermal conductivity, nuclear magnetic resonance and magnetic torque of Sb-salt 2 reveal that there is finite entropy in the very-low-temperature range, but these experimental results also reveal that there is a subtle entropy release below the liquid helium temperature (3–4 K by the specific heat capacity)^14,15,41–44^. Similar anomalous phenomena have been reported for *κ*-(ET)_2_Cu_2_(CN)_3_^20,45–48^, and these phenomena indicate that the precursor of any ordering is hidden behind the SL states. However, the ground states of *β*′-Et_2_Me_2_Sb[Pd(dmit)_2_]_2_ (Sb-salt 3), *monoclinic*-EtMe_3_P[Pd(dmit)_2_]_2_ (*m*-salt), *triclinic*-EtMe_3_P[Pd(dmit)_2_]_2_ (*t*-salt) and *β*′-Cs[Pd(dmit)_2_]_2_ (Cs-salt) are CO states accompanied by the alternation in the inter-molecular distances which is denoted as “valence bond ordering” (=VBO)^30,32–36,38–40,49^. The pressure inducing the superconducting transition of the *m*-salt is lower than that of any other X[Pd(dmit)_2_]_2_ salt exhibiting the AF ground state^24,49,50^. The 1/4-filled model is more appropriate than the effective 1/2-filled model for the X[Pd(dmit)_2_]_2_ salts exhibiting the CO states^38–40^. There are two kinds of VBO in X[Pd(dmit)_2_]_2_: the lattice distortions exhibiting the alternations in the inter- and intra-dimer transfer integrals. The electron densities and the alternations in the inter- and intra-dimer transfer integrals (=amplitudes of the VBOs) in the CO states shown in Table 1 were examined from the C=C stretching modes^37–40^. The CO state and VBO have also been observed for *κ*-D_3_(Cat-EDT-TTF)_2_ and *κ*-(ET)_2_B(CN)_4_, respectively^22,23^. Using information from infrared (IR) and Raman spectra of P-salt 1, P-salt 2, Sb-salt 1 and Sb-salt 2, we report that the absence of any ordering and a small entropy releasing of a Sb-salt 2 can be ascribed to dynamical fluctuations due to the competition between different types of CO states that are accompanied by different types of VBOs.

## Results

**Figure 2:**
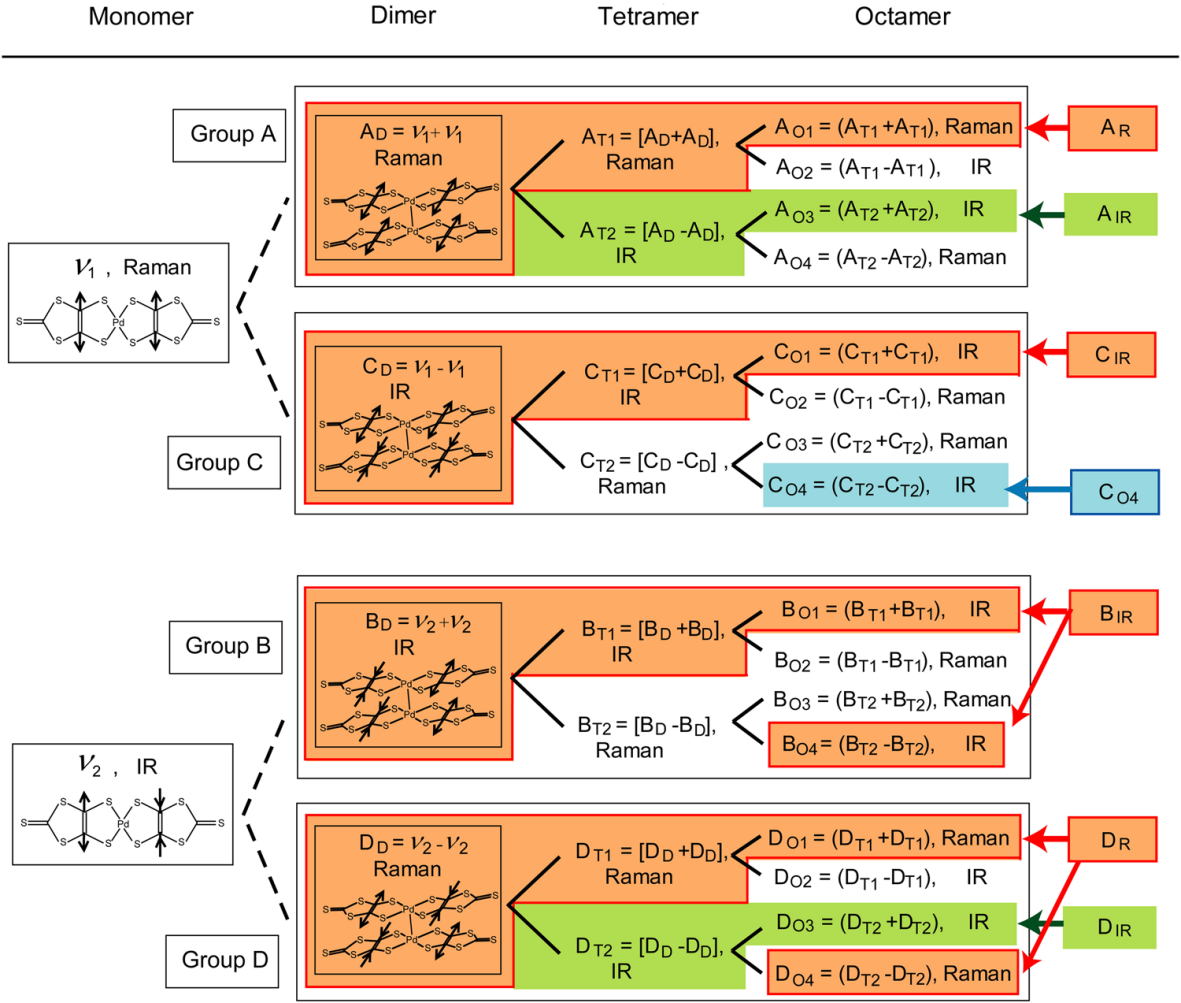
Correlation diagram of C=C stretching modes in monomers, dimers, tetramers and octamers that have centre of inversion symmetries. The tetramers and octamers are composed of two and four dimers, respectively. The C=C stretching modes of the monomers and dimers are shown by the arrows. A_D_–D_D_: C=C stretching modes of a dimer, A_T1_–D_T2_: C=C stretching modes of a tetramer, and A_O1_–D_O4_: C=C stretching modes of an octamer. The positive and negative signs for each vibrational mode denote in-phase and out-of-phase vibrations, respectively. These C=C stretching modes are classified into Groups A–D. A_R_-D_IR_ in the right column correspond to peaks in Fig. 3. C_O4_ denoted by the blue area is independent of any C=C stretching modes of tetramer and dimer. A_IR_ and D_IR_, denoted by green area, consist of C=C stretching modes of tetramer and octamer, but these are independent of any C=C stretching modes of dimer. A_IR_ consists of the A_T2_ and A_O3_ modes, and D_IR_ consists of the D_T2_ and D_O3_ modes. A_R_, C_IR_, B_IR_ and D_R_, denoted by orange area, consist of C=C stretching modes of dimer, tetramer and octamer. A_R_ consists of the A_D_, A_T1_ and A_O1_ modes, C_IR_ consists of the C_D_, C_T1_ and C_O1_ modes, B_IR_ consists of the B_D_, B_T1_, B_O1_ and B_O4_ modes, and D_R_ consists of the D_D_, D_T1_, D_O1_ and D_O4_ modes.

The electron densities and the amplitudes of the VBOs for the SL and AF salts were examined from the C=C stretching modes. Prior to discussing the experimental results for the SL and AF salts, we show how C=C stretching modes of monomers, dimers, tetramers and octamers are observed (Fig. 2). Hereafter, the word dimer will always refer to a dimer that has a chemical bond between monomers. We assume that the dimers, tetramers and octamers in Fig. 2 each have a centre of inversion symmetry. When a 2D layer is composed of dimers, none of the C=C stretching modes, except for the C_D_ and B_D_ modes, should be observable in an IR spectra. Furthermore, none of the C=C stretching modes, aside from those for the A_D_ and D_D_ modes, should be observable in a Raman spectra. This mutual exclusion rule can be applied not only to equilateral lattices but also to isosceles and scalene lattices when there is no VBO in a 2D layer. With regards to tetramers, the number of C=C stretching mode becomes eight (from A_T1_ to D_T2_), all of which belong to Groups A–D in Fig. 2. The total number of C=C stretching modes in an octamer is sixteen (from A_O1_ to D_O4_), all of which also belong to Groups A–D.

**Figure 3:**
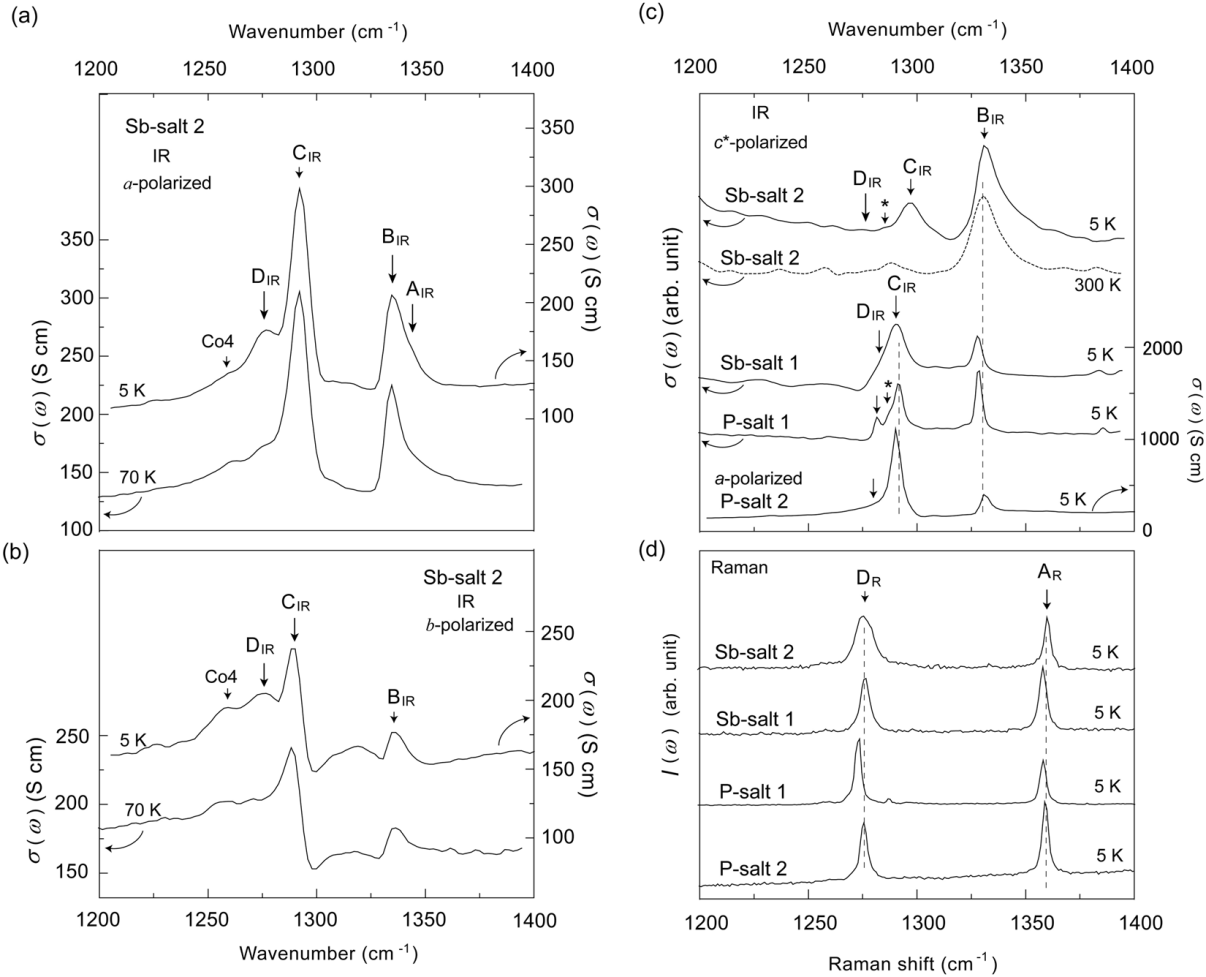
IR and Raman spectra. (**a**) *a*-polarized IR spectra for Sb-salt 2. (**b**) *b*-polarized IR spectra for Sb-salt 2. (**c**) c*-polarized IR spectra of P-salt 1, Sb-salt 1 and Sb-salt 2. The *a*-polarized IR spectra of P-salt 2 is also shown in (**c**). (**d**) Raman spectra observed at 5 K. Aside from Sb-salt 2, all of the salts here have antiferromagnetic ground states. All of the IR spectra were obtained as conductivity spectra from the Kramers–Kronig transformation of the corresponding reflectance spectra.

Figure 3(a)–(d) show the Raman spectra and the conductivity spectra in the IR region for P-salt 1, P-salt 2, Sb-salt 1 and Sb-salt 2. Hereafter, the IR spectra designate the conductivity spectra in the IR region. The notations of the C=C stretching modes in Fig. 3 correspond to those in the right column of Fig. 2, and the frequencies of the C=C stretching modes are summarised in Table 2. More than two C=C stretching modes were observed in the IR spectra of Sb-salt 2 (Fig. 3(a)), which indicates that its lattice was composed of tetramers or octamers. The C=C stretching modes for Sb-salt 2 can be assigned based on the IR and Raman spectra of Sb-salt 3, Cs-salt, *t*-salt and *m*-salt, where all of the dimer modes (from A_D_ to D_D_) were split into multiple peaks due to the occurrence of tetramers and octamers^37–40^. The Raman and IR spectra in the CO states are shown in Supplementary information^37–40^. C_O4_ shown in Fig. 2 is easily distinguishable from any other C=C stretching modes because its frequency is the lowest due to the electron-molecular vibrational (e-mv) interaction involving both inter- and intra-dimer charge transfers^37,40^. The C_O4_ mode is characteristic of an octamer and independent of the C=C stretching modes belonging to a dimer and a tetramer. We have found that the C_O4_ mode for Sb-salt 2 in the *a*- and *b*-polarized spectra (Fig. 3(a) and (b)); however, its intensity was significantly weak in comparison to those of the Cs-salt and Sb-salt 3 exhibiting static octamers shown by Supplementary information^37,40^. This result indicates that there should not be any static CO state due to the octamers and the octamers exhibits fluctuation not only at 70 K but also at 5 K.

**Table 2:** Frequencies of the C=C stretching modes.

Salt	A_R_ (cm^−1^)	A_IR_ (cm^−1^)	B_IR_ (cm^−1^)	Δ*B* (cm^−1^)	C_IR_ (cm^−1^)	C_O4_ (cm^−1^)	D_R_ (cm^−1^)	D_IR_ (cm^−1^)
P-salt 1	1358.1	—	1328.4	3.3	1291.1	—	1273.0	1281.5
P-salt 2	1358.8	—	1331.4	4.4	1290.2	—	1275.5	1280.0
Sb-salt 1	1357.9	—	1327.9	4.8	1290.1	—	1276.1	1282.6
Sb-salt 2 (5 K)	1359.9	1342.9	1331.7	10.1	1296.9	1259	1275.7	1276.7
Sb-salt 2 (300 K)	1356.3	—	1330.6	—	1288.2	—	1265.3	—

A_R_, A_IR_, B_IR_, C_IR_, C_O4_, D_R_ and D_IR_ correspond to those in Figs 2 and 3. Δ*B* denotes the linewidth of B_IR_. D_IR_, A_IR_, C_O4_ and Δ*B* were obtained from the curve fitting analyses.

D_IR_ was observed in both the *a*- and *b*-polarised spectra at 5 K (Fig. 3(a) and (b)). Because the linewidth is large, *i*.*e*. 16 cm^−1^, D_IR_ consists not only of the D_T2_ mode of a tetramer but also of the D_O3_ mode of an octamer, those which are denoted by the green area in Fig. 2. These spectral features support that both the tetramers and octamers exhibit fluctuations in the SL state. The modes of D_IR_ were observable at 70 K, which indicates that the fluctuation remains at this temperature. The D_IR_ mode is also observed in the c*-polarized spectra although its intensity is significantly weak. This phenomena is ascribed to the fact that the long axes of the [Pd(dmit)_2_]_2_ molecules are not exactly normal to the *ab*-plane^37–40^. We have found that D_IR_ is commonly observed in the c*-polarised spectra of P-salt 1, P-salt 2 and Sb-salt 1. This result suggests that the fluctuation due to forming tetramer or octamer is non-negligible for the AF salts, *i.e*. P-salt 1, P-salt 2 and Sb-salt 1. In particular, the IR and Raman spectra of P-salt 1 exhibit several minor vibrational modes, which indicates the phase separation. To investigate the inhomogeneous charges and the inter-molecular interactions in the AF salts, further experiments including the *a*- and *b*-polarized spectra are required.

A_IR_ was observed as a shoulder of B_IR_ in the *a*-polarized spectra at 5 K (*i.e*. Sb-salt 2 (Fig. 3(a)). As shown in Fig. 2, A_IR_ consists of A_T2_ in a tetramer and A_O3_ in an octamer, those which are denoted by the green area. The frequency of A_IR_ (1342.9 cm^**−**1^) was found to be significantly lower than that of A_R_ (1359.9 cm^**−**1^) in Fig. 3(d). This difference in the frequencies is due to the inter-dimer transfer integral in a tetramer being larger than that between tetramers^38,39^. The intensity of A_IR_ was very weak in comparison to that of B_IR_. Conversely, the intensities of A_IR_ were comparable to those of B_IR_ in the CO states, *i.e*. in *t*-salt and *m*-salt^38,39^. The weak intensity of Sb-salt 2 indicates that it does not include any static tetramers or octamers; in other words, fluctuations in the formation of both octamers and tetramers take place in the SL state.

The linewidth of D_R_ in Fig. 3(d) of Sb-salt 2 is larger than that of any salt that had an AF ground state. These spectral features indicate the fluctuation in the formation of the octamers and tetramers in the SL state of Sb-salt 2. When octamers, tetramers and dimers compete with one another, the frequencies of D_D_, D_T1_, D_O1_ and D_O4_ denoted as the orange area in Fig. 2 are close to each other in the Raman spectra, which leads to the broadening of D_R_. However, neither peak separation nor broadening was observed in the D_R_ mode in the AF ground states. This result indicates that the fluctuation was greater in Sb-salt 2 than in P-salt 1, P-salt 2 and Sb-salt 1. The degree of fluctuation is quantitatively discussed in the Discussion section.

The vibrational modes at 1331 cm^−1^ in Fig. 3(a)–(c) belong to B_IR_. This assignment is supported by the relative intensities in the c*-polarised spectra being higher than those in the *a*- and *b*-polarised spectra^37–40^. The frequency of B_IR_ is proportional to the molecular charge^40^. The frequencies in the AF states of P-salt 1, P-salt 2 and Sb-salt 1 reveal that all of the monomer charges are equal to −0.5, *i.e*. [Pd(dmit)_2_]^0.5−^. The frequency of Sb-salt 2 also indicates that it consists of [Pd(dmit)_2_]^0.5−^; however, the linewidth of Sb-salt 2 shown as Δ*B* in Table 2 is twice or three times as large as Δ*B* of the AF salts. This result indicates the inhomogeneous molecular charge in the SL state of Sb-salts 2. As shown in Supplementary information, Group B in the CO state exhibits the peak separation and the separated peaks belong to the charge-rich and charge-poor molecules^37–40^. Δ*B* in the CO state is obtained from the difference in the frequencies^37–40^. Δ*B* of Sb-salt 2 is smaller than those in the CO states of Sb-salt 3, Cs-salt and *t*-salt (37, 43, 35 cm^−1^, respectively), but a slightly larger than that in the CO state of *m*-salt (6 cm^−1^). B_IR_ of Sb-salt 2 does not exhibit any peak separation whereas that of *m*-salt exhibits the peak separation due to the static tetramer (B_T1_ and B_T2_)^38^. The absence of any peak separation and the broad linewidth for Sb-salt 2 indicate that an inhomogeneous molecular charge distribution in the SL state is ascribed to the fluctuation due to forming octamer, tetramer and dimer. Thus, B_IR_ in the SL state of Sb-salt 2 consists of B_D_, B_T1_, B_O1_ and B_O4_, those which are denoted as the orange area in Fig. 2.

As shown by the orange areas in Fig. 2, A_R_ and C_IR_ in Fig. 3 consists of the C=C stretching modes for dimer, tetramer and octamer. As described in the previous literatures and Supplementary information, the frequencies of A_R_ and C_IR_ (=C_T1_ and C_O1_) are insensitive to the change in the inter-molecular interaction. Thus, the inter-molecular interaction cannot be analysed from A_R_ and C_IR_^37–40^.

## Discussion

**Figure 4:**
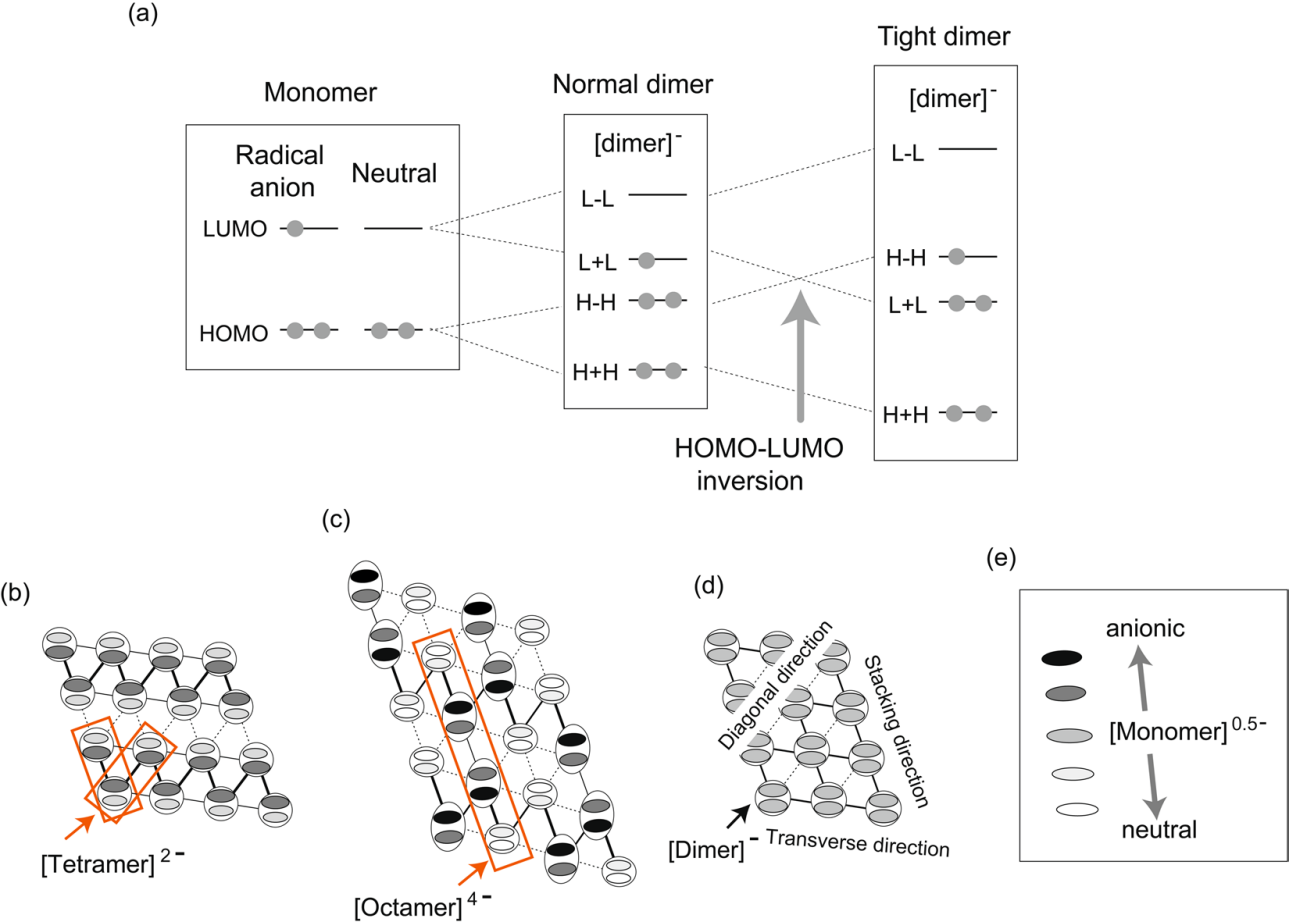
HOMO–LUMO inversion, and charge distributions and bond alternations in a 2D layer of X[Pd(dmit)_2_]_2_. (**a**) Energy diagram of the monomers, normal dimer, and tight dimer. “L” and “H” represent the LUMO and HOMO of a monomer, respectively. The sum (+) and subtraction (−) between them designate bonding and antibonding interactions, respectively. (**b**) and (**c**): Two of three different CO states revealed by our previous works. (**d**): Layer consisting of regularly arranged [dimers]^−^. (**e**): Legend of electron densities of monomers in (**b**–**d**). The black, grey and white sections in the monomers of (**b**–**d**) denote the electron densities in the HOMO of (**b**) [tetramer]^2−^, (**c**) [octamer]^4−^ and (**d**) [dimer]^−^. Different patterns of the inter-dimer bond alternations; the bold, thin and dotted lines between the dimers denote the strong, intermediate and weak inter-dimer interactions, respectively, between the HOMOs in (**b**) [tetramer]^2−^ and (**c**) [octamer]^4−^. The interacting monomers that form tetramers or octamers in (**b**) and (**c**) are highlighted by the orange rectangles. Inter-dimer VBOs in (**b**) are formed along two of three directions in the triangular lattice. Intra-dimer charge separation in (**b**) is accompanied by inter-dimer VBOs. The intra-dimer interaction in a charge-rich dimer of (**c**) is weaker than that in a charge-poor dimer, which manifests itself as the intra-dimer VBO. Inter-dimer VBOs are also formed in (**c**). Intra- and inter-dimer charge separations of (**c**) are accompanied by inter- and intra-dimer VBOs, respectively. The combination between intra- and inter-dimer VBOs is formed along one of the three directions. There is no VBO in (**d**).

The behaviour of the C=C stretching modes of Sb-salt 2 at 5 K indicates the competition between the octamers, tetramers and dimers. The CO states accompanied by the tetramers and octamers would be favourable if there were an inversion in the energy levels of the molecular orbitals near the Fermi level *ε*_F_^31,35,36,38–40^. As a result of such an inversion, the highest occupied molecular orbitals (HOMOs) and the lowest unoccupied molecular orbitals (LUMOs) of the monomers in Fig. 4(a), respectively, constitute the LUMO and HOMO of the dimer, those which are denoted as H**−**H and L + L in Fig. 4(a) ^27–29^. The interchange in the energy levels happens when the dimerization is tight enough for it to seem as if there is a chemical bond between the monomers; this is called a HOMO–LUMO inversion^24,28^. VBO due to the electron-phonon interaction is enhanced in the HOMO of [tetramer]^2−^, and the charge separation due to the nearest neighbour Coulomb repulsions (=*V*) is enhanced in the next HOMO of [tetramer]^2−^ ^38,39^. Similarly, VBO and the charge separation due to *V* are enhanced in the different orbirals of [octamer]^4−^ near the Fermi level, *ε*_F_^31,32,36^. As a result, VBO and *V* originating from the different orbitals are cooperatively enhanced in the CO state of the HOMO–LUMO inversion system. On the other hand, the CO states, spin liquid and superconductivity of the BEDT-TTF salts are ascribed to the cooperation between VBO and *V* in the same HOMOs^8^. However, there is no theoretical model on the cooperative interaction involving different orbitals. The enhancement of both VBO and *V* in the X[Pd(dmit)_2_]_2_ salts results in several kinds of CO states being accompanied by bond alternations, two of which are shown by Fig. 4(b) and (c) ^30,37–40,51^. The CO states shown by Fig. 4(b) and (c) are accompanied by tetramers and octamers, respectively. The 2D layer containing tetramers shown in Fig. 4(b) exhibits a difference in the inter-dimer transfer integrals^38,39^. This difference is referred to by this paper as an inter-dimer VBO. The 2D layer containing octamers shown in Fig. 4(c) exhibits a difference in the intra-dimer transfer integrals^30,40^; this difference is referred as an intra-dimer VBO. The 2D layer of Fig. 4(c) also exhibits the inter-dimer VBO^30,40^. For each CO state, the ionic molecules are next to each other due to the cooperation between VBO and *V*. Figure 4(d) shows the 2D layer under the assumption that X[Pd(dmit)_2_]_2_ belongs to a Mott insulator, where there is no inter-dimer or intra-dimer VBO and there is no CO. On the other hand, the effective 1/4-filled model rather than the effective 1/2-filled model is applicable to a tetramer and an octamer^38–40^. In this Discussion section, the magnitudes of the intra-dimer and inter-dimer VBOs are examined in the SL state (*i.e*. Sb-salt 2), and they are compared to those of the AF states (P-salt 1, P-salt 2 and Sb-salt 1). The quantitative analyses of the intra-dimer and inter-dimer VBOs can be done using the A and D groups, but cannot be done using the C group because the frequency of the C_O4_ is extraordinary perturbed by both inter-dimer and intra-dimer charge transfers^37,40^.

To begin with, we examine the intra-dimer VBO. Because the frequency of the D_R_ mode decreases as the magnitude of dimerization increases^37,40^, the peak separation or broad linewidth in the D_R_ mode indicates the inhomogeneity in the dimerization. The degree of inhomogeneity is estimated from Δ*D*, which is defined as being the difference in the frequencies of the D_R_ modes of the charge-rich and charge-poor dimers (D_O1_ and D_O4_ in Fig. 2) ^37,40^. When the D_R_ mode does not show any recognisable peak separation, Δ*D* is defined as being the linewidth of D_R_^40^. Table 3 shows Δ*D* in the AF, SL and CO states. Δ*D* in the AF state becomes large when *T*_AF_ is small. Δ*D* of Sb-salt 2 is larger than that of any salt containing AF ground states. These experimental results indicate that the inhomogeneity of the dimerization increases from the AF to the SL states. This conclusion is inconsistent with that of a previous report, in which the dielectric constant was found to have no obvious relationship with *T*_AF_^52^. This inconsistency suggests that the anomaly in the dielectric constant should include not only the inhomogeneity due to the bond alternations but also macroscopic inhomogeneities. However, Δ*D* of Sb-salt 2 is smaller than Δ*D* of the octamers in the CO states (Sb-salt 3 and Cs-salt), and it is of an intermediate value and comparable to that of Cs-salt at 100 K and *t*-salt in the CO state. Cs-salt above *T*_CO_ exhibits a dynamical fluctuation due to the competition between the octamer and tetramer, and *t*-salt in the CO state contains residual octamers in the 2D layer consisting of tetramers^39,40^. Therefore, the intermediate value of Δ*D* of Sb-salt 2 indicates the fluctuation of the intra-dimer bond alternation.

**Table 3:** Amplitudes of intra-dimer and inter-dimer bond alternations and charge inhomogeneity.

Material	Δ*D* (cm^−1^)	Δ*A* (cm^−1^)	δ*D* (cm^−1^)	Δ*ρ*	References	Ground state
P-salt 1	(3.4)	—	8.5	(0.05)	This work	AF: *T*_AF_ = 42 K^21^
P-salt 2	(4.0)	—	4.4	(0.06)	This work	AF: *T*_AF_ = 17 K^21^
Sb-salt 1	(5.1)	—	6.5	(0.07)	This work	AF: *T*_AF_ = 16 K^21^
Sb-salt 2	(9.5)	17	(16)	(0.14)	This work	SL
*m*-salt	(4.9)	23	—	0.09	^38^	CO: *T*_CO_ = 20 K^49,50^
*t*-salt	(9.5)	27	—	0.5	^39^	CO: *T*_CO_ = 50 K^39^
Sb-salt 3	46	11–20	—	0.52	^37,40^	CO: *T*_CO_ = 70 K^30^
Cs-salt	15	29	—	0.72	^40^	CO: *T*_CO_ = 56 K^29^
Cs-salt (100 K)	(9.3)	29	—	0.13	^40^	—

Δ*D* denotes the amplitude of the intra-dimer bond alternation; Δ*D* values with parentheses correspond to the linewidth of the D_R_ mode, and Δ*D* values without parentheses correspond to the difference in the frequencies between the D_O1_ and D_O4_ modes. Δ*A* and δ*D* denote the amplitudes of the inter-dimer bond alternations; Δ*A* is defined by the difference in the frequencies between the A_R_ and A_IR_ modes. δ*D* values without parentheses are defined by the difference in the frequencies between the D_IR_ and D_R_ modes, while δ*D* values with parentheses correspond to the linewidth of D_IR_. Δ*ρ* refers to charge inhomogeneity, and the Δ*ρ* values with parentheses were estimated from the linewidth of the B_IR_ mode, *i.e*., ΔB in Table 2. Δ*ρ* values without parentheses were estimated from the difference in the frequencies of the Group B belonging to charge-rich and charge-poor molecules. Δ*ρ* in the CO states of Sb-salt 3 and Cs-salt denote the charge inhomogeneity between the most charge-rich and the most charge-poor molecules.

Next, we examine the inter-dimer VBO. The magnitude of the bond alternation in tetramers is defined as being Δ*A*, which is the difference in the frequencies between the A_R_ (=A_T1_) and A_IR_ (=A_T2_) modes^38,39^. The Δ*A* value of the octamers is reflected in the alternation between the bold line and the thin line in the rectangle in Fig. 4(c) ^40^. Δ*A* of Sb-salt 2 is not larger than any of the values of Δ*A* in Table 3. This result suggests that the inter-dimer VBO is not evident in the SL state, which is in agreement with the fact that the A_IR_ mode in the SL state is weak.

However, Δ*A* cannot be obtained from the IR and Raman spectra of the AF salts (P-salt1, P-salt 2 and Sb-salt 1), because the A_IR_ mode is not observed clearly in the IR spectra. Nevertheless, the frequencies of D_IR_ are slightly higher than those of D_R_. When the 2D layer contains tetramers, D_R_ exhibits the symmetric vibration with respect to centre of inversion symmetry in a tetramer whereas D_IR_ exhibits asymmetric vibration. D_R_ induces the inter-dimer charge transfer whereas D_IR_ does not. This relationship is comparable to that between A_IR_ and A_R_. Similarly to Δ*A*, the magnitude of the bond alternation in tetramers is proportional to the difference in the frequencies between D_R_ and D_IR_. The frequencies of D_R_ and D_IR_ in the AF salts were estimated from the Raman and c*-polarized spectra, respectively. The difference in the frequencies (δ*D*) is shown in Table 3. Non-zero δ*D* indicates the inter-dimer VBO in the AF salts. Nevertheless, both δ*D* and Δ*D* are small for the AF salts. Any accurate value of D_IR_ cannot be obtained from the c*-polarized spectra of Sb-salt 2 because its intensity is very weak and the linewidth is large. The frequency and linewidth were obtained from the *a*-polarized spectra. Nevertheless, no accurate values of δ*D* (D_IR_ **−** D_R_) could be obtained for Sb-salt 2, because the linewidths of both D_R_ and D_IR_ are broad (D_R_ = 9.5 and D_IR_ = 16.4 cm^**−**1^). The broad linewidth of D_IR_ suggests that there is an inhomogeneity in the inter-dimer VBO; thus, the linewidth can be regarded as being the maximum value in the magnitude of the bond alternation. This value is shown in Table 3 as δ*D* for Sb-salt 2. This estimation is supported by δ*D* being almost identical to Δ*A*. The δ*D* of Sb-salt 2 is significantly larger than that of any of the AF salts (P-salt1, P-salt 2 and Sb-salt 1); therefore, the magnitude of the inter-dimer VBO in the SL state is intermediate between the AF and CO states. Although the δ*D* of the AF salts are small, the observation of the D_IR_ mode in the c*-polarized spectra indicates the weak charge inhomogeneity due to the dynamical or static fluctuation.

Table 3 shows the inhomogeneity in the molecular charges. The difference in the molecular charges between the charge-rich and charge-poor molecules (*i*.*e*. Δ*ρ* of [Pd(dmit)_2_]^(0.5±Δ*ρ*)**−**^) can be estimated from Δ*B* ^40^. Δ*ρ* in the SL and AF states were obtained from this work, and Δ*ρ* in the CO states were obtained from the previous experiments^37–40^. Δ*ρ* of Sb-salt 2 is larger than those in the AF states (P-salt 1, P-salt 2 and Sb-salt 1), a slightly larger than that in the CO state of *m*-salt and smaller than those in the CO states of t-salt, Sb-salt 2 and Cs-salts. Δ*ρ* of Sb-salt 2 is comparable to that of Cs-salt at 100 K, where tetramers and octamers are competing with each other. This result supports the dynamical fluctuation in the SL state of Sb-salt 2.

The inter-dimer transfer integrals of X[Pd(dmit)_2_]_2_ are too small for metallic bonds; rather, inter-dimer interactions induce tetramers and octamers. As a result, Δ*A* (and δ*D*) and Δ*D* become non-zero. In a tetramer, Δ*A* (and δ*D*) increases, but Δ*D* remains small; in an octamer, not only does Δ*A* (and δ*D*) increase but so does Δ*D*. δ*D* and Δ*D* decrease in the AF salts. As for Sb-salt 2, both Δ*A* (and δ*D*) and Δ*D* are intermediate between the AF state and octamer. Quantitative analyses of the electron-molecular vibration coupling modes in the SL state indicate that the dynamical fluctuations are due to the competition among three building blocks; octamers, tetramers and dimers. The broad linewidth in Group B supports the motional narrowing due to the dynamical fluctuation among the octamers, tetramers and dimers. These spectral features indicate that the solid solution consisting of three building blocks exhibits the fluctuation; *i*.*e*. 4[dimer]^−^ ↔ 2[tetramer]^2−^ ↔ [octamer]^4−^ ↔ 4[dimer]^−^. No ordering in Sb-salt 2 can be ascribed to the fluctuation in the triangular lattice. In the SL state of Sb-salt 2, all of the charge distribution patterns in Fig. 4(b,c) and (d) participate in the fluctuation. Because of the fluctuation, no ordering is considered to be realised, even though the 2D layer slightly deviates from that of an equilateral triangular lattice. Without the dynamical fluctuation, a subtle deviation from the equilateral triangular lattice should produce a CO state accompanied by VBO, which was the case for *m*-salt and Sb-salt 3^32,34–38,40,49,50,53^. The broad and small peak in the normalized *C*_p_ *T* ^−1^ vs *T* plot obtained from the specific heat measurement of Sb-salt 2 is in agreement with the fluctuation involving the tetramers and octamers because the tetramers and octamers induce the small entropy releasing^43^. The fluctuation is also consistent with the fact that Sb-salt 2 retains the finite entropy in the very low temperature region.

## Conclusion

We have examined the inter-dimer bond alternation, intra-dimer bond alternation and inhomogeneous molecular charges of Sb-salt 2, which we believe to be a quantum SL. The vibrational spectroscopies that focused on the C=C stretching modes revealed that the dynamical fluctuation was due to the competition among the dimers, tetramers and octamers in the SL state. A HOMO–LUMO inversion was found to favour several kinds of CO states consisting of tetramers and octamers, which enhanced the dynamical fluctuation and the inhomogeneous charges. Therefore, the SL state of Sb-salt 2 is ascribed to the dynamical fluctuation. We also have found that an inhomogeneous molecular charge accompanied by bond alternation is non-negligible in the AF states.

## Methods

Single crystals of P-salt 1, P-salt 2, Sb-salt 1 and Sb-salt 2 were synthesised by aerial oxidation from the acetone solution of [(CH_3_)_4_P]_2_[Pd(dmit)_2_], [(C_2_H_5_)_2_(CH_3_)_2_P]_2_[Pd(dmit)_2_], [(CH_3_)_4_Sb]_2_[Pd(dmit)_2_] and [(C_2_H_5_)(CH_3_)_3_Sb]_2_[Pd(dmit)_2_], respectively^17^. The thickness of the single-crystal of Sb-salt 2 was *ca*. 10 μm, and the area of the 2D plane (*i.e*. the crystallographic *ab*-plane) was *ca*. 200 × 200 μm^2^. The *a*- and *b*-polarised IR-reflectance spectra of Sb-salt 2 were measured at the Institute for Molecular Science (IMS) using a Thermo Nicolet Nexus 8700 equipped with a SpectraTech IR-Plan Microscope. The *b*-direction corresponds to the transverse direction in Fig. 1(c) and (d), while the *a*-direction is perpendicular to the transverse direction. The *a*-polarised IR-reflectance spectra of P-salt 2 were also measured at IMS. The c*-polarised IR-reflectance spectra of P-salt 1, Sb-salt 1 and Sb-salt 2 were measured at Beamline No.43IR in SPring-8 using Bruker IFS120HR, since the spatial resolution of the equipment (10 × 10 μm^2^) was suitable for measurements on the crystal edge. However, the absolute reflectivity could not be obtained. The conductivity spectra were obtained by a Kramers–Kronig transformation from the IR-reflectance spectra. The Raman spectra of P-salt 1, P-salt 2, Sb-salt 1 and Sb-salt 2 were measured at the IMS using a Ramascope (Renishaw) with the backward scattering configuration. The wavelength of the incident light was 633 nm from the He-Ne laser, which was suitable for observing the C=C stretching modes. Samples used to measure both the IR-reflectance and Raman spectra were cooled using a He-flow cryostat. The detailed experimental set-up for *m*-salt, *t*-salt, Sb-salt 3 and Cs-salt has been reported elsewhere^37–40^.

## Electronic supplementary material

Supplementary Information
