# Supplementary material for: Charge and Lattice Fluctuations in Molecule-Based Spin Liquids
Source: Sci Rep. 2017 Oct 10;7:12930. doi: 10.1038/s41598-017-13118-4 (PMC5635065; doi:10.1038/s41598-017-13118-4)
Supplement: Supplementary file 1 — Supplementary Information [file 41598_2017_13118_MOESM1_ESM.pdf]

Supplementary Information: Charge and Lattice Fluctuations in Molecule-Based Spin Liquids

Takashi Yamamoto,[1,2*] Takashi Fujimoto,[1] Toshio Naito,[1] Yasuhiro Nakazawa,[3] Masafumi Tamura,[4] Kyuya Yakushi,[5] Yuka Ikemoto,[6] Taro Moriwaki,[6] & Reizo Kato[2]

[1] Graduate School of Science and Technology, Ehime University, 2-5 Bunkyo-cho, Matsuyama, 7908577, Japan

[2] RIKEN, 2-1 Hirosawa, Wako, 3510198, Japan

[3] Graduate School of Science, Osaka University, 1-1 Machikaneyama, Toyonaka, Osaka 560-0043, Japan

[4] Department of Physics, Faculty of Science and Technology, Tokyo University of Science, 2641 Yamazaki, Noda, 2788510, Japan

[5] Toyota Physical and Chemical Research Institute, 41-1 Yokomichi, Nagakute, 4801192, Japan

[6] JASRI, SPring-8, 1-1-1 Kouto, Sayo, Hyogo 679-5198, Japan

Correspondence and requests for materials should be addressed to T.Y. (email: yamataka@ehime-u.ac.jp)

C=C stretching modes in the CO states of t-salt, m-salt, Sb-salt 3 and Cs-salt [1-4].

Figures S1-S4 show the Raman spectra, and *c**-, *a*- and *b*-polarized IR-conductivity spectra. Abbreviations and experimental temperatures are appended to all spectra. The Raman spectra obtained by the 514 and 633 nm lasers are shown by the green and red curves, and the Raman spectra obtained by 785 nm lasers are shown by the black and purple curves, respectively. The incident lights, except for the purple curve, were normal to the edge of the single crystals. The incident light of the purple curve were normal to the largest plane of the single crystal. The *c**-polarized spectra of Cs-salt was not observed because the thickness of a single crystal was lower than 10 μm. The assignments of t- and m-salts are based on the C=C stretching modes of a tetramer, *i.e.*, A_{T1}~D_{T2} modes in Fig. 2. The assignment of Sb-salt 3 and Cs-salt are based on those of an octamer, *i.e.*, A_{O1}~D_{O4} modes in Fig. 2. The broken and straight lines are guides for the eyes. The broad peaks denoted by the broken curves belong to the C Group, those which are perturbed by the electron-molecular vibrational (e-mv) interaction. Most of the symmetric and asymmetric modes shown by Fig. 2 are observed in the Raman and IR-conductivity spectra, respectively. The A_{O2} modes of Cs-salt and Sb-salt 3, those which inherently belong to the asymmetric modes, are observed in the Raman spectra because of the Resonance effect. The observation of both B_{T1} and B_{T2} modes in the IR-conductivity spectra of t- and m-salts is ascribed to the fact that the unit cell contains two conducting layers separated by the layer of counter cations. Although the C_{T2} modes of t- and m-salts inherently belong to the symmetric mode, those are observed in the *a*- or *b*-polarized spectra. This phenomenon is attributed to the Fano resonance, where C_{T2} is resonated by the charge transfer transition (= charge gap) in the mid-infrared region. Several C and D modes in Figs S3 and S4 are observed as the anti-resonance modes because of the Fano resonance. These anti-resonance modes are resonated with the C_{O4} or C_{T2} modes. Because the long axis of $[Pd(dmit)_2]$ is not exactly normal to the conducting and 2D layer, the C=C stretching modes do not always exhibit the polarization dependence in the IR-conductivity spectra. Detailed assignments and properties of the C=C stretching modes are described in the previous literatures. [1-4]

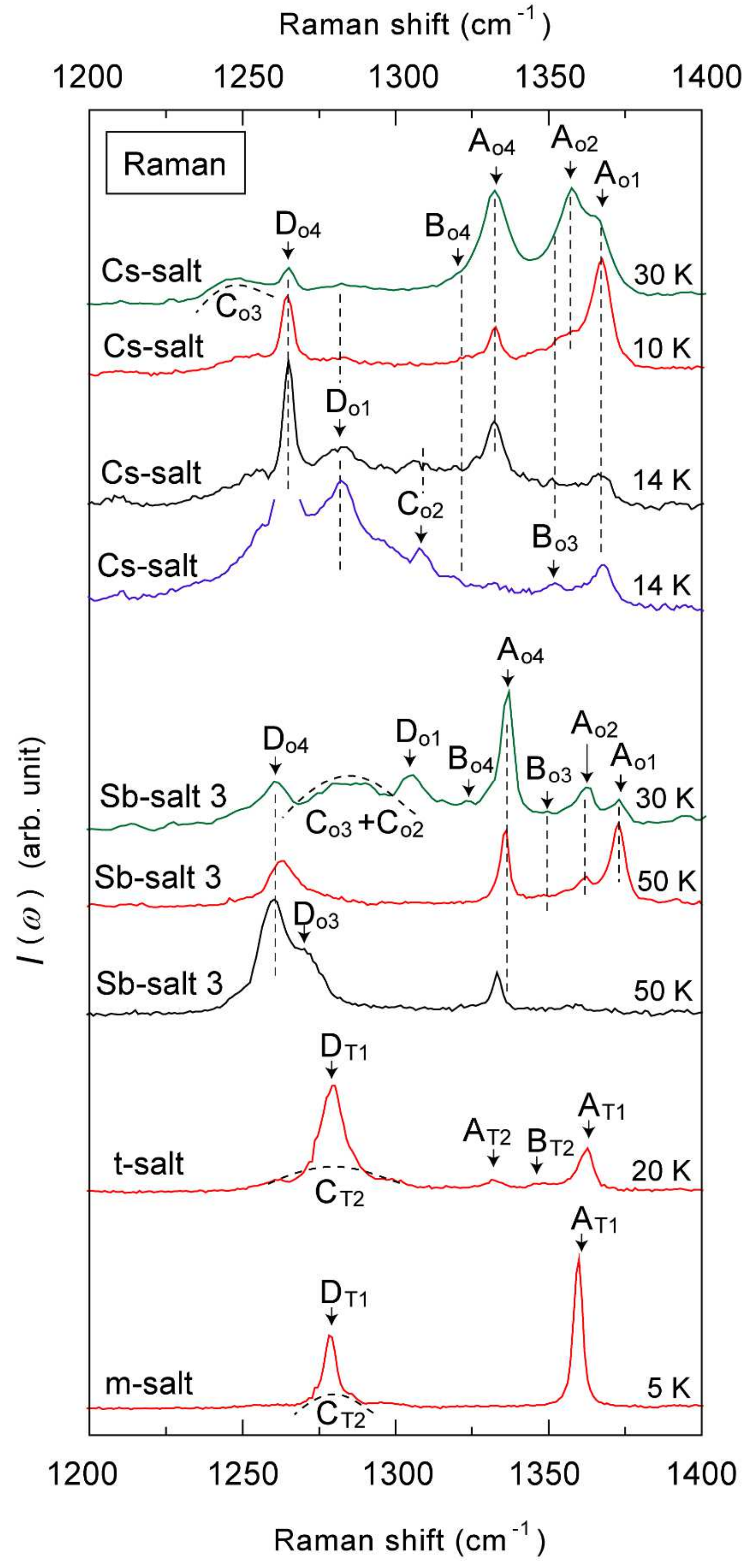

Supplementary Figure S1. Raman spectra in the CO states [1-4].

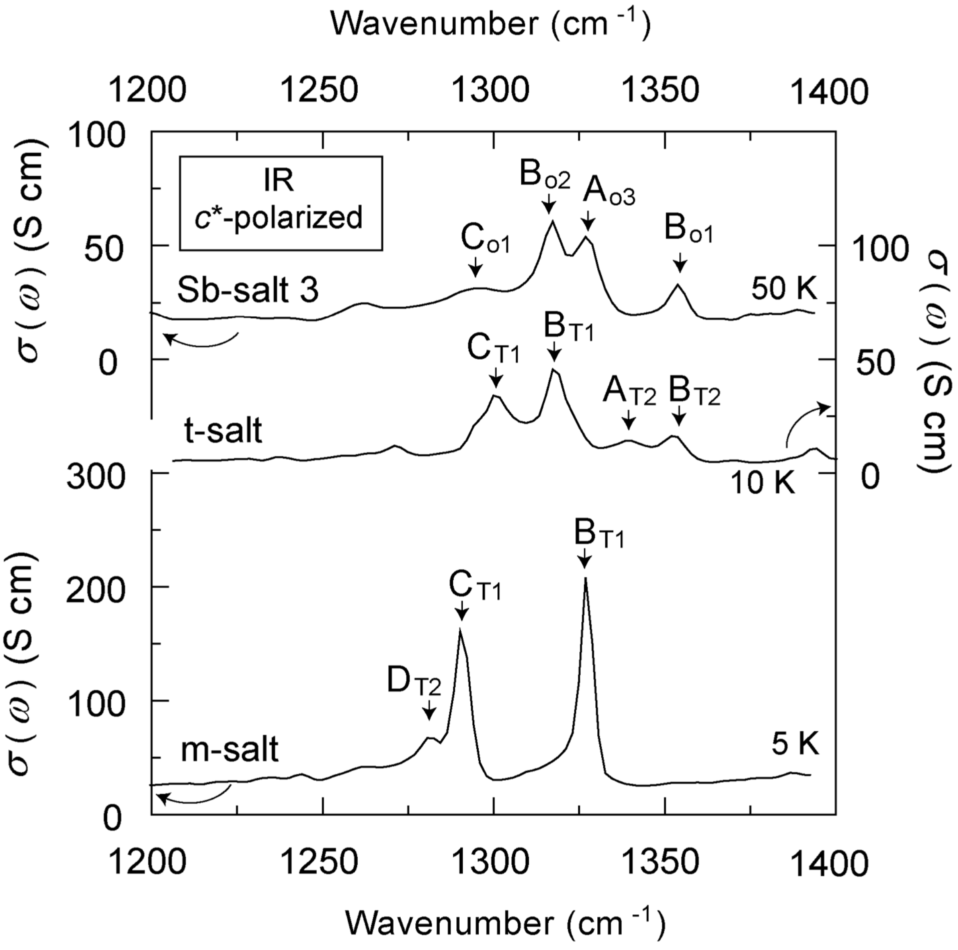

Supplementary Figure S2. *c**-polarized IR-conductivity spectra in the CO states [1-4].

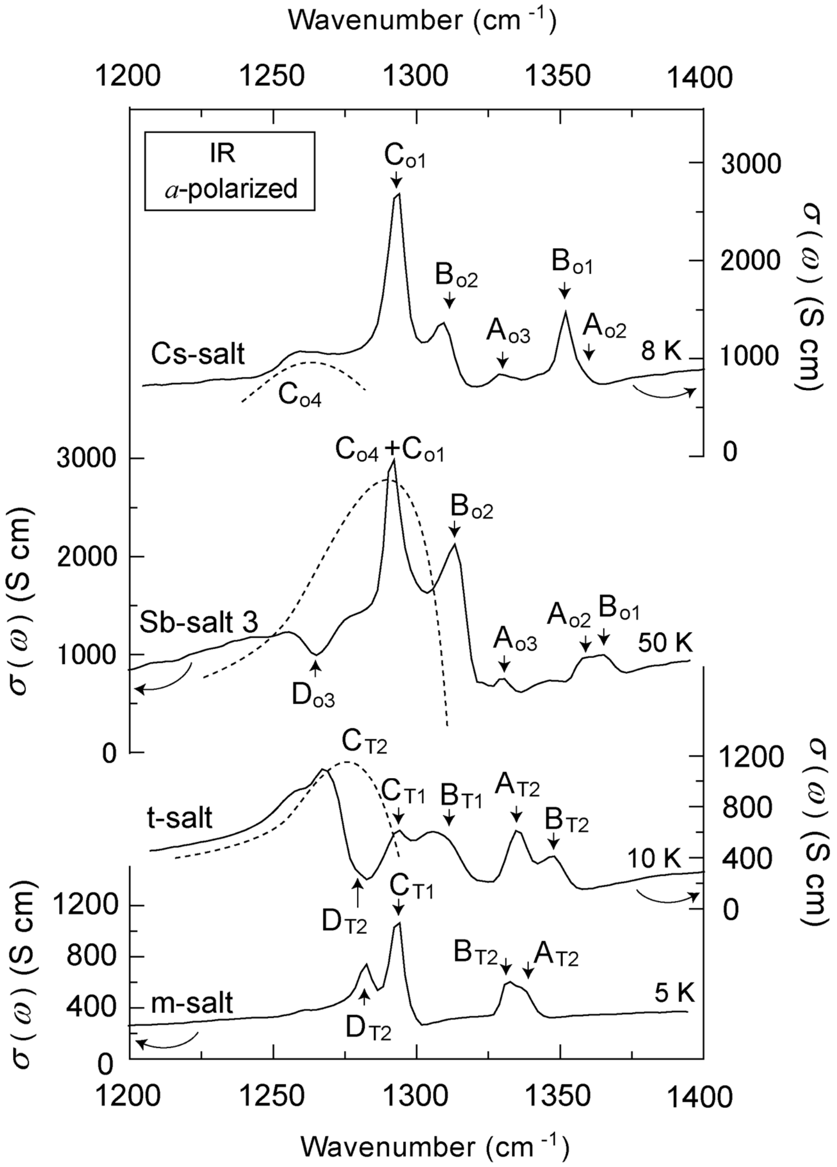

Supplementary Figure S3. *a*-polarized IR-conductivity spectra in the CO states [1-4].

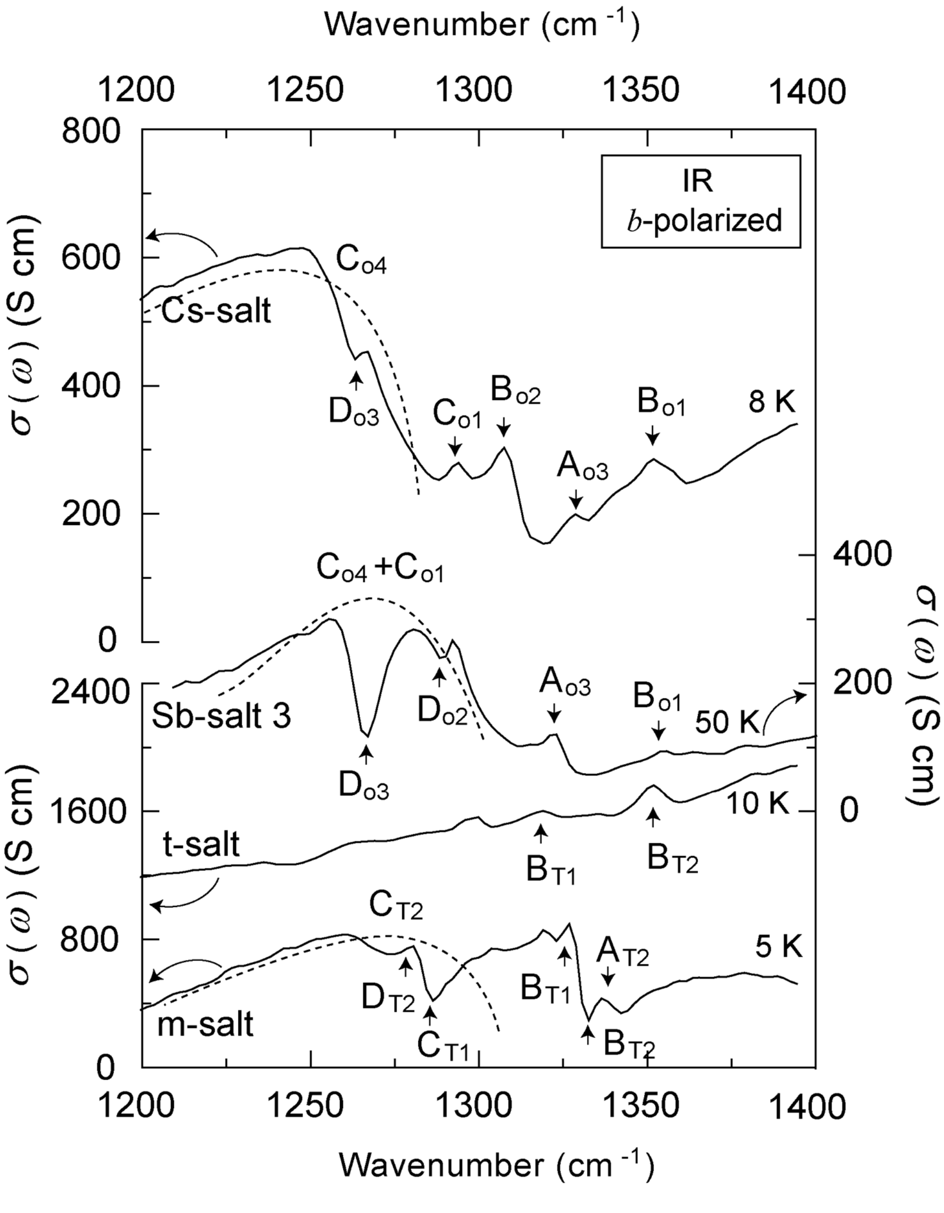

Supplementary Figure S4. *b*-polarized IR-conductivity spectra in the CO states [1-4].

References

1. Yamamoto, T. *et al.* Vibrational spectra of $[Pd(dmit)_2]$ dimer (dmit= 1, 3-dithiole-2-thione-4, 5-dithiolate): Methodology for examining charge, inter-molecular interactions, and orbital. *Journal of the Physical Society of Japan* **80**, 074717 (2011).
2. Yamamoto, T. *et al.* Property of the Valence-Bond Ordering in Molecular Superconductor with a Quasi-Triangular Lattice. *Journal of the Physical Society of Japan* **83**, 053703 (2014).
3. Yamamoto, T. *et al.* Intradimer charge disproportionation in *triclinic*-$EtMe_3P[Pd(dmit)_2]$*2* (dmit: 1, 3-dithiole-2-thione-4, 5-dithiolate). *Journal of the Physical Society of Japan* **80**, 123709 (2011).
4. Yamamoto, T., Tamura, M., Yakushi, K. & Kato, R. Intra- versus inter-dimer charge inhomogeneity in the triangular lattice compounds of *β′*-$Cs[Pd(dmit)_2]_2$ and *β′*-$Et_2Me_2Sb[Pd(dmit)_2]_2$: A degree of freedom specific to an interchange of energy levels in the molecular orbitals. *Journal of the Physical Society of Japan* **85**, 104711, doi:org/10.7566/JPSJ.85.104711 (2016).
